# Observations on an Amaurosis and Cophosis, Attended with Diminution of Voice, Motion, &c. in Consequence of Organic Lesion of Several Parts of the Brain

**Published:** 1811-06

**Authors:** A. Leveque Lafource


### *Observations on an Amaurosis and Cophosis, attended with diminution of voice, motion, &c. in consequence of organic lesion of several parts of the Brain.* By A. Leveque Lafourcé, M. D.

A. DEVILLE, a seamstress, aged 35 years, born in the department of La Somme, of robust parents, of a sanguine bilious temperament, had never been attacked with any other disease than the small-pox, when in June 1808, she was seized with severe head-ache, accompanied with anorexia, giddiness, disturbed sleep, and frightful dreams. Nothing was done. Soon afterwards, giddiness was so frequent that she was obliged to keep her bed. The head-ache became severe and continued 30 days. Her sight, at first, was slightly affected, but this weakening increased sensibly with a sensation of pricking and distention at the bottom of the orbit; (a beginning of amaurosis.)

In September, 1808, the patient entered into a hospital, where she remained four months. She became worse, her sight, which had been gradually diminishing, was now entirely lost on her left side, and forty-five days after, in the right eye also; the amaurosis was now complete. The sensation of pricking and fulness at the bottom of the orbit, which, before the loss of sight was troublesome, now ceased, but the head-ache remained. Deville had for some time experienced a noise in the ear, and soon the loss of hearing on the left side was added to blindness. A cautery was used, and different internal means were employed without any benefit. The patient returned home.

In February, 1809, she was affected with general weakness; and soon afterwards a difficulty and finally incapacity in walking. She remained in this state for several months.

At the beginning of August she was carried to the Hotel-Dieu, they gave her two purges and applied a blister to the nape of the neck; these means, however, were not successful.

By the 5th of September the pains in the head had almost ceased: The face was bloated, there was stupor, continual debility in the superior and lower extremities; the pupils were much dilated, and the blindness appeared irremediable.

On the 10th, there was cophosis on the left side, and occasionally, difficulty of hearing on the right side. Her voice was altered, the tongue was inclined towards the left side. The faculties of the mind were little interrupted, except the memory, which was treacherous. She had a cough without expectoration; she could lie easy on either side, but her power of motion was very limited both from paralysis, and on account of her great weakness.

15th of September. Sleep lasted longer than in the natural state. The pulse was small, weak, and rather frequent.

25 and 26. She could hardly open her mouth; somnolency and coma succeeded, involuntary dejection, and laborious breathing, and on the following day she died. Catamenia ceased only in September.

*Dissection.*—The state of the brain: The lateral ventricles contained 2 oz. of serosity; the pituitary gland was twice as large as usual, and contained several puriform spots with a fœtid smell; it adhered strongly to the posterior clinoid processes, these, but chiefly the left, were nearly destroyed, the sella turcica and its membranous covering were in a similar condition. The sphenoidal membranes were filled with serosity mixed with pus.

In the left lateral and middle cavity of the cranium, was a tumour which had originated in the bottom of the meatus auditorius internus, and occupied three fourths of the posterior surface of the petrous portion of the temporal bone. The orifice of this meatus was destroyed, and in its place was a cavity which was filled by the tumour. The tumour was of fourteen lines in diameter, and $3\frac{1}{2}$ drams in weight, its structure partly fibrous, and partly carcinomatous. A very thin layer of dura mater covered it, it reached to the tentorium, and pressed against the annular protuberance, which it pushed about three lines out of its situation to the right.

*Reflections.*

*Reflections.*—May not the cause of the blindness, of the deafness, and of the other phænomena, be thus explained?

1st. The blindness from caries of the sphenoidal bone, and the communication of the sphenoidal membranes with the brain. The external air would have penetrated into the cranium if it had not been hindered by the pituitary gland, which it caused to inflame; and this gland mechanically closed the opening which the caries had made; and as it exceeded its usual rise and projected as far as the optic foramen, it must have made pressure upon the optic nerves, and from this pressure resulted the paralysis of the retina, and loss of sight.

2d. As to the loss of hearing, the tumour which occupied a portion of the meatus auditorius internus, had not destroyed the auditory and facial nerves, but only compressed them. The pressure must chiefly have acted on the first, and hence resulted the deafness on that side.

3d. We may explain the universal paralysis from the pressure on the annular protuberance by the aforesaid tumour; a pressure which more or less interrupted the influence of the brain upon the spinal marrow.

